# Developing a Social Responsibility Pilot Tool to Reduce Helicopter Research and Improve Social Benefit: A Cleanup Site Case Study

**DOI:** 10.1177/11786302261461687

**Published:** 2026-06-16

**Authors:** Elise M. Smith, Anna-Barbara O’James, Stephen Molldrem, Lance Hallberg, Cíntia Liara Engel

**Affiliations:** 1 Institute for Bioethics & Health Humanities, School of Public and Population Health, University of Texas Medical Branch, Galveston, TX, USA; 2 School of Public and Population Health, University of Texas Medical Branch, Galveston, TX, USA; 3 Environmental & Public Health Education and Management, Department of Pharmacology and Toxicology, University of Texas Medical Branch, Galveston, TX, USA; 4 Faculty of Social Sciences, Federal University of Goiás, Goiânia, Brazil

**Keywords:** remediation, environmental management, ethics, social responsibility, superfund sites, cleanup sites, qualitative research

## Abstract

Environmental cleanup processes take place after contamination and possible human exposure to a range of pollutants. Researchers often contribute significantly in developing knowledge to assist remediation efforts; however, their work has sometimes been critiqued as “helicopter research” that does not benefit communities. This study aimed to develop a social responsibility tool that promotes the social impact of research conducted at cleanup sites. We used a qualitative case study approach to understand perspectives of stakeholders working at a contaminated site called Jones Road in Houston, Texas. Data collection included ongoing participant observation at community events and semi-structured stakeholder interviews, consisting of fifteen exploratory interviews followed by nine interviews to validate the tool. We analyzed interview data using both inductive and deductive approaches to design a preliminary social responsibility tool that was later refined and validated. Our tool is intended to help researchers map out stakeholder needs, develop goals that align with those needs, identify their responsibilities to effectively engage with and benefit stakeholders, and evaluate social benefits. Notably, we provide reflexive questions to assist researchers to enable advocacy, share expertise, develop knowledge, manage the harms of science, ensure safety, increase transparency, and ultimately build lasting relationships.

## 1. Introduction

In response to contamination stemming from urbanization and industrialization, a range of national and international policies, legal instruments and regulatory frameworks have been developed to guide remediation planning, development and implementation.^1,2^ In the United States (U.S.), the Superfund program identifies sites prioritized for cleanup based on state or federal standards; such sites generally constitute an imminent and substantial danger to public health or the environment.^3^ The Comprehensive Environmental Response, Compensation, and Liability Act (CERCLA), passed by the U.S Congress and more commonly known as the “Superfund Act”,^4^ empowers the federal government to order responsible parties to complete removal and remedial actions and establishes substantial penalties for noncompliance. The Superfund Act aimed to expedite cleanups; however, in reality policy implementation has been a slow and inefficient process due to issues that vary from site to site.^5,6^ A lack of effective community involvement has resulted in a disconnect between environmental health perceptions and actual health improvements.^7^ Critics have argued that cleanup processes are limited or inadequate and have negative consequences on the environment and human health for communities.^8^

Scientific studies extend beyond the parameters of relevant policies and statutes, to draw attention to the identification of certain sites or help move cleanups along. In some instances, cleanup processes have been justified and initiated following the collaborative work of community groups and scientists that established evidence of potential exposures and harmful outcomes. This was exemplified in the Love Canal case in Niagara Falls, New York, where the Hooker Chemical Corporation dumped waste into the canal from 1920 to 1953.^9^ Contaminants were then covered, and the property was sold to be further developed as a residential area which included a school. Initially, residents had little recourse to deal with chemical odors emanating in the area. That is, until the Love Canal Home Association teamed up with Dr. Beverly Paigen to analyze interviews and survey data of local community members. Results of the study concluded that the area had higher than normal rates of miscarriages, nervous breakdowns, urinary tract disorders, hyperactivity, and epilepsy.^10^ The scientific evidence substantiated community claims that exposures had adverse health impacts and subjected residents to environmental injustices.

Leveraging interdisciplinary scientific expertise can play a significant role in improving cleanup assessment, planning, procedures, and outcomes.^11^ However, researchers have often tailored their work in accordance with the environmental health priorities set by funders, institutions, government and the scientific community;^12^and, these may not consider or align with priorities of resident communities. Researchers have been criticized for conducting “helicopter research,” focused narrowly on developing scholarly knowledge without input from local communities.^13^ Without due consideration of the needs of communities, it is more likely that research will be of limited benefit to local communities.^14^ When helicopter research practices yield little or no expected benefit, a participating community may feel used or exploited. Helicopter research is generally considered colonialist and unethical and leads communities to mistrust scientists.^13,15-17^

Researchers in environmental health have developed reporting back practices designed to provide greater autonomy for participants to decide what they would like to do with the information.^18^ Unfortunately, these are not widely implemented.^18^ Reasons for this shortcoming include lack of expertise and financial support, barriers regarding Institutional Review Board (IRB) approvals as well as the lack of established reporting back approaches.^18,19^ Although reporting back might assuage certain concerns about helicopter research, research goals may still not align with the needs or interests of the community and therefore, may have limited benefit. Scholars identify absences or gaps in knowledge that would benefit communities as “undone science”.^20,21^ Systemic inequities and the politics of knowledge shaped by and reflected in the distribution of power structures have explained these epistemic absences that often serve to marginalize and deter grassroot social organizations and other advocacy organizations.^21^

A broader more substantive notion of social responsibility is needed to ensure genuine social benefit and reduce helicopter research. Although many thinkers uphold the idea of integrating social responsibilities in scientific work,^22-24^ how these responsibilities should be operationalized in proactive ways remains insufficiently defined.^24^ There is general agreement in the applied ethics scholarly literature that scientists have the negative obligation to avoid causing harm, whereas the positive obligation to create benefits is far more contested.^25,26^ Debates persist over what kinds of benefits are expected,^24^ who ought to receive them,^27^ and what mechanisms, if any, can effectively hold researchers accountable for producing them.^28^ This ambiguity is consequential in the context of Superfund-related research, which carries an explicit translational mandate and has a potential to yield public health, policy and economic benefits.^29^ Research infrastructures related to Superfund research are equipped with community engagement cores which aim to facilitate the use of research findings by affected communities that ultimately leads to social benefits.^30^

The idea that science should be transformative is also central to environmental justice (EJ) which constitutes both a grassroots social movement and an interdisciplinary field of scholarly research.^31^ Founders of EJ focused on clearly describing disproportionate burdens put on communities of color focusing on race and class.^32-34^ The field later expanded to also consider broader conceptions of intersectional spaces related to gender, land and indigeneity.^35-38^ After describing disparities in EJ communities, research approaches such as action research and community-based participatory research have promoted a transformative ethos with the aim of achieving change.^39-41^ EJ scholars often used such approaches to develop work that is community focused and yields social benefits. Such work has increased research quality^42^ and improved health literacy amongst communities.^43^ However, the ability for research to impact communities in a substantial way remains limited. For example, a critical interpretive review of EJ participatory research revealed that only 26 out of 154 case study articles resulted in outcomes that impact structural change.^44^

This paper proposes a pilot tool for social responsibility that enables researchers to increase social benefits stemming from research projects conducted at cleanup sites. Specifically, we used a case study approach to explore stakeholders’ perspectives regarding the potential or real benefits and harms of different types of scientific input or evidence developed at the site. While this tool is not a generalizable model, it may serve as a starting point to design a more widely applicable instrument through further refinement, adaptation, and testing at different cleanup sites.

## 2. Methodology

A qualitative case study approach allowed us to understand stakeholders’ perspectives regarding social responsibilities of researchers at Jones Road Groundwater Plume site in Texas. In health sciences, case studies may be used with participatory approaches to generate research results that facilitate future implementation.^45^ We adopted a constructivist and pragmatic epistemological stance,^46^ recognizing that multiple subjective realities may be constructed from different stakeholder perspectives while also applying a pragmatic approach that allows us to select methods that best answer our research question.^47^ This research was reviewed by the University of Texas Medical Branch Institutional Review Board (Protocol #23-0041).

The research proceeded with a three-phase process which included: 1) Stakeholder interviews and exploration of social responsibilities, 2) development of the pilot tool based on empirical data and 3) refinement of the pilot tool. Not only does this three-phase process allow exploration, tool-building and further refinement, but it also creates a process that builds trust between researcher and participants.^48^ Recurring discussion with stakeholders in different community meetings and then in multiple subsequent interviews helped build the trust and willingness required to exchange more in-depth information. This process also allowed the research team to gain the necessary information to design a tool that participants could further refine to better align to stakeholder needs and adapt to site conditions and as such, facilitate translation into practice.

### 2.1. Phase One: Stakeholder Interviews and Exploration of Social Responsibilities

In phase one, we conducted fifteen semi-structured interviews with stakeholders involved in the Jones Road Groundwater Plume Superfund site to understand their perceptions regarding potential or actual benefits and harms of different types of scientific input or evidence developed at the site. We developed a convenience sample that included stakeholder groups with knowledge of the history of the site and who had been directly or indirectly impacted over time. We used the concept of “information power” developed by Malterud and colleagues to justify the sample size.^49^ According to this approach, the larger the information power the sample holds, the lower the number of participants is needed. Information power is linked to study aim, sample specificity, use of established theory, quality of dialogue and analysis strategy.^49^ We consider our information power to be very high since our study was case-specific, based on a limited population (individuals impacted by this Superfund site) and also applied theory related to social responsibility which we previously developed.^25^ Moreover, using both interviews and participant-observation allowed for in-depth accounts of different stakeholders. In accordance with Malterud and colleague’s approach, we re-evaluated the sample throughout our research process to ensure sufficient information was collected.

Stakeholders included community members who live in or around the neighborhood, non-profit workers who have worked to disseminate knowledge, public health workers who have helped in the remediation process, scientists conducting research at the site and political representatives who advocate for the community. These groups were chosen because they are the main players who influence the remedial process and are present at public meetings to discuss relevant site matters. Participants needed to be 18 years of age or more and speak English to participate in the study. Stakeholders publicly involved in the site were recruited via email and flyers distributed at community events. Stakeholders were asked to share the flyers with individuals who fit our inclusion and exclusion criteria. Compensation included a $30 gift card for the first interview and a $15 gift card for the second interview.

To understand the context of research, we completed participant observation at 14 public events where non-profits provided updates about the site to the community which included ongoing discussion about the scientific project, the results and next steps. These observations were crucial to understanding the relational dynamics between stakeholders and interpreting the data from the interviews. Researchers attending the meetings took detailed memos throughout and after the event. We used the community meetings to give contextually relevant examples during the interview process.

The open-ended interview guide for phase one was developed based on general categories from our prior work that identified three core dimensions of social responsibility: (1) relevance, (2) usability and (3) sustainability.^25^ We also asked general questions about the stakeholder’s knowledge of the site, how it impacted them and if they believe that scientists had a social responsibility to mitigate environmental harms or injustices. We included examples from the community events to further contextualize the questions being asked and give more substance to the conversations. The interviews were transcribed and anonymized.

### 2.2. Phase Two: Development of Pilot Tool Based on Empirical Data

During phase two, analysis of the qualitative data involved integration of deductive and inductive coding.^50,51^ For the deduction section of the analysis, we used the three core dimensions in the questionnaire. Given the short-term nature of the scientific research project being conducted at Jones Road we did not focus on sustainability, we focused on relevance and usability. The deductive step was followed by inductive coding to further explore the idea of relevance and usability while also adding additional core dimensions that researchers would suggest. Our inductive approach was based on Schreier’s Qualitative Content Analysis which is heavily influenced by Grounded Theory since it centers open coding but remains unique in that it doesn’t aim to result in a full theory with explanatory power.^51^ Our open coding included conceptualizing the material to generally understand what is happening followed by a definition of categories which was done by grouping similar concepts together, and further developing and refining of categories. The codes were developed inductively by three members of the team, who met weekly to discuss and refine the codes to ensure consistent interpretation. To validate the coding framework, five different transcripts were coded by at least two coders independently. The team discussed disagreements about the coding framework and was able to arrive at an agreement. The rest of the interviews were coded using the agreed upon framework.

Based on the themes mentioned by stakeholders, we then developed a decision-making tool that promotes social responsibility. To do so, the research team considered the themes related to social responsibility criteria and developed reflexive questions linked to the themes. The team deliberated ideas about the process over a two month period constantly going back to the transcripts and codes.

### 2.3. Phase Three: Refinement of the Pilot Tool

During phase three, we conducted a second round of interviews with participants that had also participated in the previous round of interviews. Attrition resulted in a final sample of nine participants for the second round interviews. During this interview phase we presented the tool on a PowerPoint slide deck and explained each step of the process. We aimed to refine the tool to gain practical usability and relevance. Interviews lasted thirty minutes to one hour and were recorded since meaning from visual interactions were useful. We integrated all modifications suggested by the stakeholders in the tool.

## 3. Results

### 3.1. Mapping Stakeholders in the Jones Road Case Within a Context of Uncertainty

The Jones Road Groundwater Plume site originated at the former Bell Dry Cleaners facility located within a Shopping Center beyond the city limits of northwest Houston, Texas. The former Bell facility practiced improper disposal of dry-cleaning products including tetrachloroethylene (PCE) from 1988 to 2002. There is evidence that PCE and its derivatives are carcinogenic and neurotoxic to humans and can impact their nervous, immune and hematologic systems as well as impact human reproduction and development.^52^ In September of 2003, the site was listed on the National Priorities List (NPL) following the detection of PCE in several nearby private wells.^53^ Since the responsible party - Bell Dry Cleaners facility - no longer owned the business, the Jones Road Site was deemed an orphan site requiring the US Environmental Protection Agency (EPA) to take responsibility for the cleanup process.

The EPA directed the installation of water line connections to residences and businesses at the site to provide the community with an alternative source of drinking water. These connections would be related to the waterline managed by the White Oak Bend Municipal Utility District (MUD). Although the connections would be free of charge, there would be a charge for future use of water. Despite efforts to inform community members of risks related to PCE, many residents have continued to use their contaminated well water^54^ in order to avoid any additional fees typically associated with out-of-district MUD water. The EPA enacted long-term remedial measures include plugging the wells and In-Situ Enhancements to Pump and Treat which entail treating the soil and groundwater without extraction, groundwater and indoor air monitoring.^55^ In a 2022 report, the EPA stated that the site was still “not protective”.^56^ Actions required to make the site protective include preventing exposure to contaminated wells and evaluating the vapor intrusion pathways to ensure that they do not contain concentrations of contaminants of concern (COC) that exceed allowable cleanup levels.^56^

Texas Health Environmental Alliance (THEA), a 501c(3) non-profit organization, has been proactive in keeping residents informed by facilitating community meetings. THEA partnered with the University of Texas Medical Branch (UTMB) in 2023 to assess the extent of contamination migration from the site’s epicenter beyond the boundaries of EPA’s purview. Community members have actively participated in this collaborative study, with their wells sampled and tested for COC.^57^ Findings from independent sampling of 13 wells revealed elevated levels of COC from the dry-cleaning facility. According to discussions with UTMB researchers conducting the study, two well samples out of the 55 analyzed revealed the presence of COC and there was no evidence of the plume shifting. THEA’s upcoming initiatives include the examination of health surveys amassed over numerous years, as well as partnering with the Texas Department of State Health Services to evaluate the region for potential clusters of cancer and other prevalent diseases possibly linked to the Jones Road Superfund site.

In the context of this research, each stakeholder had a different role as outlined in Table 1. Table 2 groups participants recruited according to demographic characteristics. All stakeholders were represented in the first round of the interviews. Due to attrition, the second interview round included a smaller sample. It was particularly challenging to recruit policy members and public health workers where discussions about EJ were disallowed in the workplace or politically compromising. Alternative types of recruitment strategies would be needed to focus on these stakeholders in future research.

**Table 1.** Stakeholder Mapping

Stakeholders	Phase 1 interviews	Phase 3 interviews	Role at jones road
Scientists	4	3	• Developed research to assess plume boundaries
			• Provided talks about the presence and impact of contamination
Non-profit workers	3	3	• Created public events to discuss cleanup progress
			• Provided information about Superfund site to the public
Community member	6	3	• Attended events
			• Impacted directly and indirectly by cleanup activities
Policy maker	1	0	• Created pressure on the EPA to ensure safety for residents
Public health worker	1	0	• Involved in the cleanup process and development

**Table 2.** Demographics of Participants in the Study

Demographic variables	Phase 1 interviews (N=15)	Phase 3 interviews (N=9)
Gender		
Female	9	7
Male	6	2
Race		
American Indian/Alaskan Native	0	0
Asian	1	0
Black/African American	0	0
Native Hawaiian or other pacific islander	0	0
More than one racial identity	2	1
N/A	0	0
White	12	8
I do not wish to disclose	0	0
Ethnicity		
Hispanic	4	3
Non-Hispanic	11	6

The central theme in discussions about the Jones Road situation was managing uncertainty in part because it is impossible to know how much contamination was dumped and for how long. Confusingly for many stakeholders, the EPA kept changing the information presented at public meetings regarding the site: “at one point the EPA had a meeting and said the plume had moved. (…) Then the next meeting they had, they said no, it had never moved. So, I think people were like, what? You know, which is it?” (ID10) The non-profit workers mentioned that lack of research or lack of certainty regarding presence of contamination was often interpreted as a reason not to pursue further remediation that could keep people safe. Faced with changing and uncertain information, reaching out to alternative sources like an independent researcher seemed important to the non-profit. Many stakeholders including scientists mentioned that in depth scientific details might not always be needed. One scientist mentioned: “I don’t think at this point the community is asking for beyond a shadow of a doubt certainty. They want to know what is the probability that might be the case and then how can they use that information to make decisions” (ID6). In fact, scientific details may make the general impact of the site on human health more confusing to community members. Contrary to the EPA that requires high levels of certainty and scientific information to act, other stakeholders could apply a different level of certainty and still generate social benefits from activities like education and risk prevention, further discussed in this paper.

### 3.2. Social Responsibility: Making Science Relevant and Useful to Stakeholders

Social responsibility can be defined as research that is relevant and useful to stakeholders in order to improve health outcomes or reduce disparities.^25^ Participants were asked to identify what is relevant and useful scientific information in the case of Jones Road. We define relevant research as that which addresses or responds to the needs of at least one actor with the goal of creating some sort of social benefit. The usefulness of that research is determined by access to knowledge in understandable form, its cost of application and complexity of use. A review of definitions are available in Table 3.

**Table 3.** Definitions and Examples of Relevant, Usable and Uncertain Science

Code	Definition	Example
Relevance of science	•Research is relevant to the needs of at least one stakeholder with the goal of creating social benefit. The social benefit may include reducing disparities, improving community or public health or improving individual health.	•Relevant research may include contributions from a discipline of research that is particularly important (e.g. hydrology, toxicology). Relevance may also include research that is aligned to the needs of groups of community members.
Usability of science	•Usefulness of research including access to knowledge, costs of application and complexity of use. Complexity may refer to the differing values or priorities that influence the use of users.	•Usable research may include research that is accessible, relatable and applied to provide solutions to the problems.
Uncertain science	•Managing the lack of sureness about science, data, knowledge, or implications of knowledge. Uncertainty may range from being completely unsure about the situation to falling short of certainty. This implies the unwillingness to believe in conclusive evidence regarding the topic that is being discussed.	•Uncertain science includes all discussions regarding limitations of science, uncertainty of outcomes or implications or science.

Scientists suggested that the most relevant and useful information conveyed knowledge about plume movement and reports of any chemicals of concern as they arise. Scientists also mentioned that importance of understanding biological relevance of the chemicals present so that community can more fully appreciate the health risk resulting from contamination. Non-profit workers had a much broader vision than scientists and highlighted how it is also relevant to understand the “big picture” (ID13) using interdisciplinary expertise and different kinds of data collection (e.g. health data, contamination level data) that can help in understanding the past, present and future impacts of exposure. One non-profit worker highlighted how community members want to know “why they should care about it” (ID2).

When asking the community members what scientific information is most relevant, they often focused on information that explained the health impacts of the site and the likelihood that contamination had caused, or could cause, past and future health issues or deaths. One community member mentioned that understanding the timeline of events to assess whether the contamination levels are changing would be relevant. Another mentioned the relevance of knowledge about how the chemicals travel through aquifers. Many members of the community mentioned that scientists are relevant because they educate the public, who are often misinformed about the Superfund site. The public health worker and the policy maker mentioned that it is relevant for stakeholders to have general information to understand what is going on and how to keep themselves safe. The policy maker mentioned that it is relevant for different actors to understand “the level of danger, level of toxicity, how they can be preventive in their own household, or on their own property” (ID8).

When asked what is most useful in knowledge development, scientists suggested that additional scientific equipment for faster more frequent water testing would make data collection more efficient. Scientists also mentioned that individual reports and community reports were useful tools which create significant social impact. According to non-profit workers, scientific evidence or data becomes most useful when it is novel and not already available by other actors, such as the EPA, the Texas Commission on Environmental Quality (TCEQ) or local municipalities. This information can help in suggesting solutions to better protect communities from health issues related to contamination.

Community members mentioned that the most useful information could serve in legal proceedings or in reports presented to local representatives with the authority and power to change things. Many community members mentioned that there should be more accessible information to adequately educate the community about the Superfund site before diving into novel work. According to the PH worker, scientific information is most useful when it becomes personal and facilitates one-on-one discussion, or when it is used to engage people in collective action to reach their goals. The policy maker suggested that it is useful to let people understand how they can protect themselves by testing their own water sources and avail themselves of alternatives to stay safe. The policy maker highlighted that it is important that the community recognize that policy makers and representatives are engaged and vocal about the issues and do exert pressure on responsible agencies.

In summary, relevance and usefulness of information are defined as related to each stakeholder’s position and it can meaningfully benefit them. Producing relevant and useful science requires understanding what each stakeholder needs and how they can apply the information in their specific context. An overview of the perceptions of different stakeholders are provided in Table 4.

**Table 4.** Overview of Relevant and Useful Science Based on Perceptions of Different Stakeholders

​	Relevant science	Useful science
Scientists	• Knowledge about the plume moving	• Equipment that is easy to use
	• Reporting chemicals of concern	• Individual and Community Reports
Non-profit workers	• Expertise related to past, present exposures.	• Holistic approach
		• Evidence not provided by other actors
	• Communicating “why should community care”	• Honoring agreed upon expectations (e.g. timelines)
Community members	• Cancer cluster information	• Information for litigation
		• Understandable information to engage elected officials and the public
	• If or how contaminants are linked to human health	• Faster information
		• Monitoring information
PH worker	• Responding to questions from community members based on individual circumstances	• Open conversation with all actors
		• Using science in collective action
Policy maker	• Level of danger, safety and toxicity	• Preventive measures and monitoring Simplifying everything to “what is dangerous to you”
		• Taking active part in the debate

### 3.3. Social Responsibilities – Putting Science Into Practice

The previous section looked at what scientific activities may be useful and relevant to actors. This following section will look at the responsibilities which researchers can take on to ensure that science is both relevant and usable. Responsibilities are grouped under general themes including the need to provide neutral expertise and support broad knowledge development, enabling advocacy and collective actions, managing risks and promoting safety, and upholding relational values and transparency to build trust.

#### 3.3.1. Provide Neutral Expertise and Support Broad Knowledge Development

As discussed previously, certain participants did not trust the government and by extension the EPA. Scientists were deemed to provide neutral, unbiased expertise and knowledge development with limited vested interests. In practice, non-profit workers asked specific questions of researchers for up-to-date, apolitical scientific information to best inform community members. Scientists did mention that Community Engagement Cores were formed to familiarize community with relevant sources of expertise; these cores were not intended to conduct more research but rather as a bridge between science and society.

When the scientific expertise is insufficient to understand what is happening at a Superfund site, as was the case respecting the movement of the plume at Jones Road, research is needed. The researchers working at the site also collected updated health data of people living in vicinity of the plume; scientists wanted to create new knowledge that the EPA could not develop or collect. One scientist mentioned that they “don’t want to replace EPA. EPA certainly has their own role, and they are quite good at it. It is a question of what we can do to contribute to the process. (…) I think there is a critical role that science will play in changing Superfund Sites in the future” (ID6). This scientist draws a clear distinction between the role of the EPA, which is extremely regulated and limited, the role of community, and their role as scientist. ‬It was clear during conversations that knowledge development did not aim to contradict or embarrass the EPA for not doing enough remediation. Rather, the goal was to provide scientific evidence that could help EPA justify broader remediation.

#### 3.3.2. Enable Advocacy and Collective Action

According to non-profit workers, the EPA highlights the lack of evidence as being a reason to avoid or limit additional remedial measures. In other words, stakeholders would need to provide evidence of demonstrable or impending harms to warrant further remedial actions. Non-profit workers opined that researchers could play an active role in advocacy when contributing scientific information useful to communities and non-profits obtaining and promoting remedial solutions. Although scientists generally agreed about providing data to be used for advocacy purposes, the very term “advocacy” was problematic to some scientists and might introduce an element of bias into their work. One scientist was very clear that they could not take an active role in advocacy because they were funded to conduct “objective” research.

If we are funded by the federal government (…) We are not allowed to advocate. (…) science by its nature is objective and what we can do is provide the community with enough information, with enough data, with enough evidence for them to advocate for what they feel is best (ID 6)

Another scientist suggested that they advocated when they testified in court regarding the presence of chemicals that would impact communities. At one community meeting a non-profit worker mentioned that “science alone will not change anything”. He went on to state that policy makers, non-profits, and most importantly collective movements use scientific evidence to create public pressure to effect change through representatives, the EPA or responsible parties. In sum, most actors believed that scientists could participate in advocacy by providing the right types of information in the right type of format to impact decision-making and contribute to change. However, if they are not proactively collaborating with other stakeholders, scientists feel like they have a very limited role in advocacy.

#### 3.3.3. Managing Risk of Harm and Promoting Safety

Informing the community about contamination and how to mitigate risks as soon as possible was seen as central to the timely and effective management of harm. In the case of Jones Road, participants felt that the lag between data collection and data reporting was unacceptably long and fell short of their expectations. This delay exacerbated fear of harm, which many considered as an additional harm attributable to the research itself, particularly because no measures were offered to help reduce risk:

*coming into a community saying that there are potentially harmful contaminants, and you are going and testing for them, you want to be able to get that information back to them as soon as possible so that you can kind of help mitigate the fear that you kind of stirrup when you come into a community and just make sure that people stay informed during the process* (ID2).

It is important to note that all samples collected outside of the original plume were negative for COC; had the opposite been true, the sharing of information would have been completed in an urgent fashion according to researchers. A public representative mentioned that many environmental laws are established based on risk. If a scientist demonstrates an appreciable level of risk, legal frameworks and policies may consequently be enacted to keep people safe. However, some community members did perceive scientists as opportunists who conduct research solely for their own gain. This certainly does not favor authentic dialogue or serve to keep community safe which leads to a misalignment between perceived interest of researchers and those of other stakeholders.

Safety was a topic of crucial importance in discussions with participants. Non-profit workers highlighted their desire to keep community safe. A few non-profit workers felt that the EPA had not provided sufficient information regarding safety. Most community members wanted to know how to keep themselves and their family safe. Community members had general safety-related questions such as “is it safe to drink the water?” or “is it safe for children and pets to play outside?” Some community members desired much more practical information from scientists on effective preventative methods, devices to monitor household air or regularly test water for contaminants. Testing or applying preventative measures in research was deemed to become increasingly relevant and effective as the community learned to further apply such measures.

#### 3.3.4. Upholding Relational Values and Transparency to Build Trust

Given the complexity of Superfund sites, different participants mentioned needing to rely on one another to identify knowledge gaps and determine how to address them. Non-profit workers and community members mentioned that to gain the trust of a community prior to initiating a research project, researchers should listen and learn from the local knowledge, acquire better contextual understanding and gain an appreciation of the needs of the community. Undertaking a research project without the necessary contextual knowledge could yield results of limited use to stakeholders or simply rehash already locally known knowledge.

Building a trusting relationship between local actors and researchers proved to be challenging in the case of Jones Road. Regardless of the researchers’ good intentions, participant needs were not met; actions such as a delayed data reporting timeline are one such incident. This delay not only impacted the trust of community with the scientist, it also reflected poorly on those non-profit workers who had collaborated with scientists. Non-profit workers highlighted the significant reputational risk that non-profits put on the line when they become the go-between or the messenger between researchers and the community. In response to this issue, one non-profit worker suggested that there could have been a more substantial division of labor and responsibility between the researcher and the non-profit to move the project along in a timely manner.

Matters respecting the effective communication of scientific information varied in keeping with the information needs of respective stakeholders. For example, information about contamination sent to the EPA would need to be expressed in technical evidence-based terms that meet scientific standards to be taken seriously in the remedial plan. However, most stakeholders especially members of the community prefer understandable, timely reports written in non-scientific language, which was seen as critical in maintaining transparency and obtaining the trust of community stakeholders. Case in point, community members who had their water and soil tested did eventually receive individualized results via mail. However, according to non-profit workers, the information was not understandable to community members and “made them feel stupid” (ID11). This concern was corroborated at the community meetings where members noted that the use of statistics and other technical results information like Gas Chromatography Mass Spectrometry (GC-MS) spikes detracted from an understanding of relevant information. Non-profit workers mentioned that results should have been reviewed by a lay person without a science background to determine if it was understandable to the community generally. A few participants mentioned that scientists should consider alternative venues to share information including television or radio.

Conversely, a few community members preferred to limit the communication of information so as to reduce the extent of its availability beyond community borders and avoid possible adverse impact on housing prices and the local economy. Some actors suggested tailoring the communication of results more narrowly to best align with stakeholder needs, since communicating other types of information may simply be distracting or not particularly useful. An overview of responsibilities is available in Table 5.

**Table 5.** Overview of Social Responsibilities Identified as Central by Coders

Theme	Responsibilities	Definitions
Provide Neutral Expertise and Support Broad Knowledge Development	•Expertise	•Providing training or guidance based in scientific knowledge
	•Knowledge development	•Development of new data or technology to better assess issues linked to contamination.
Enable Advocacy and Collective Action	•Advocacy	•Using science to advocate for social change. This may be done in collaboration with stakeholders, including non-profits or policy-makers.
Managing Risk of Harm and Promoting Safety	•Managing harms of science or knowledge	•Mitigating or pre-empting negative impacts (harms) coming from science or scientists.
	•Safety	•Reducing risks related to contaminants. This may include identifying contaminants of concern, risk management
Upholding Relational Values and Transparency to Build Trust	•Relational	•Promoting values linked to relationship-building which main include trust, reliability and communication.
	•Transparency	•Providing clear and complete information about the research, the researchers role, the researcher’s interest, etc.

### 3.4. Building a Social Responsibility Tool

**Figure 1.**
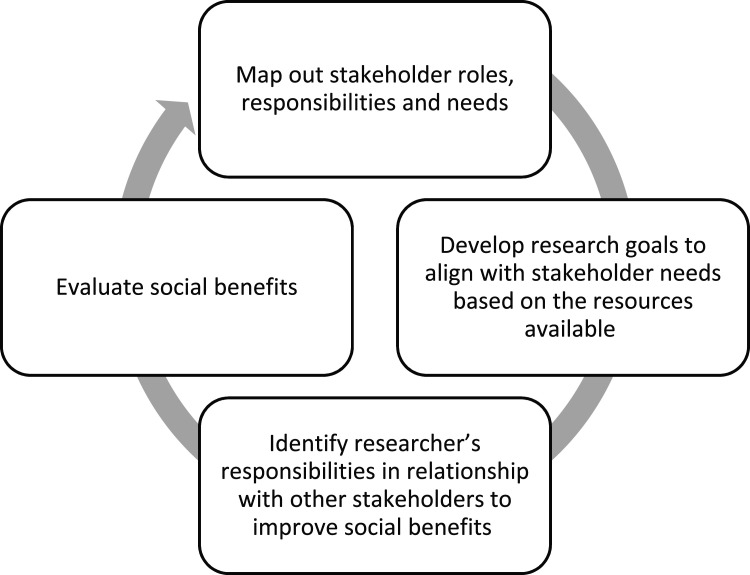
Overview of Decision-Making Tool informed and validated by stakeholders

We designed a decision-making tool to help researchers integrate social responsibilities into their research planning. To do so, the research team considered the themes related to social responsibility criteria and actions that were suggested by stakeholders. We developed a list of questions that would help researchers think of each theme in the previous section. However, we quickly realized that to uphold the relational values mentioned in the previous section (3.3.4) a process that could start before the research begins all the way throughout the research pathway would allow for better communication, trust, and accountability. Therefore, we developed a four-step process in which we embedded reflective and guiding questions related to each theme to help researcher uphold their social responsibilities. The research team then reviewed the decision-making tool with nine stakeholders to validate the feasibility and completeness of the tool. The tool was then further refined to reflect this feedback. The decision-making tool includes the four steps illustrated in Figure 1 and described further in the next section.

#### 3.4.1. Map out Participant Stakeholder Roles, Responsibilities and Needs at the Superfund Site

In the course of many interviews, it became obvious that respondents lacked an understanding of the role and responsibilities of the various stakeholders involved. For example, some community members suggested that scientists should help with remediation while others were unsure if scientists were also EPA officials. This raised unrealistic expectations for various stakeholders, including scientists, to carry out responsibilities for which they were untrained or unaccountable. To better clarify roles and responsibilities, we suggest identifying a list of stakeholders like those in Table 6. Participants repeatedly emphasized that scientists need to dedicate time listening to actors to fully understand their needs and where they are or want to be in the process or remediation. One non-profit worker mentioned that it takes time for communities to organize and decide what, if anything, they wish to do about a Superfund Site.

**Table 6.** Reflective Questions That Align With Social Responsibilities

Responsibility	Reflection questions
Uphold Relational values	• Is your relationship with other stakeholders based on trust?
	• Do you have an open channel of communication with stakeholders?
	• Have you engaged with stakeholders at the outset of the research and throughout the research process?
Enable Advocacy Initiatives	• Can you provide information or expertise that will help in advocacy causes including policy development?
Share Expertise	• Can your gained knowledge be used to guide other stakeholders in next steps to be undertaken?
Take Part in Knowledge development	• Is your research developing new data that can be used to assess issues linked to site contamination?
	• Are you using new equipment that can help inform remedial actions?
Manage harms	• Are there negative impacts related to your scientific research?
	• Have potential negative impacts been discussed with stakeholders?
Ensure Transparency	• Are you providing the community with clear and complete information about the research?
	• Is the information provided in a timely manner?
	• Should there be some delays or setbacks, are you communicating those to stakeholders?

#### 3.4.2. Develop Research Goals to Align With Stakeholder Needs Based on the Resources Available

When working on Superfund sites, it is important that researchers realize that there are very different needs and interests at play. In the case of Jones Road, the needs of Stakeholders did not seem to conflict, but they were different. Participants mentioned that in other Superfund sites where there is a responsible party that needs to cover the costs of the remediation, there may well be more conflicting needs and interests. In the Jones Road case, the research goals aligned with goals set out by the non-profit. Still, some community members would have benefitted from different types of information and research. Participants mentioned that too much time during meetings was spent talking about contamination levels as opposed to ways to mitigate health risks.

It remains essential that goals align to the extent possible with the needs of many stakeholders such that immediate as well as longer term social benefits may be realized. Clarifying goals, limitations and process of a research project with stakeholders is a pre-requisite to getting everyone on the same page. Participants suggested that integrating feedback from different stakeholders at the outset is also central to facilitating implementation and building trust. Ideally, effective and meaningful collaboration with community groups requires sharing responsibility for the research among all stakeholders. Since Superfund sites already have various actors conducting research including the EPA and non-profits, community members in the midst of data collection may well feel overburdened. It is important to situate knowledge within different initiatives to avoid redundancy and also find ways to share resources if possible.

#### 3.4.3. Identify Researcher’s Responsibilities in Relationship With Other Stakeholders to Improve Social Benefits

Since the obligations linked to social responsibility are central to our goal, this step is considered fundamental. We drew upon the themes discussed in the previous social responsibility section to formulate questions listed in Table 7 below; these are intended for researchers to consider as part of their planning processes and regular team meetings.

**Table 7.** Table of Stakeholders, Roles, Responsibilities and Needs

Stakeholders	Roles and responsibilities	Needs
Community	​	​
Non-profit	​	​
EPA	​	​
Industry	​	​
Scientist	​	​
Policy-makers	​	​
Local Business	​	​

#### 3.4.4. Evaluate Social Benefits

Our experience at Jones Road suggested that the best intentions do not always yield results and meet needs of stakeholders. Evaluating the social benefits of research and the extent to which these align with the needs of stakeholders is critical in determining their impact stakeholders. Some participants in our study suggested that this could be done using different modalities including interviews and surveys, while other participants gave the caveat to not unduly contribute to or exacerbate responder burnout. Participants did suggest that self-evaluation could be sufficient in some cases, or consulting a select number of representatives may suffice.

## 4. Discussion

Our Jones Road Superfund case study highlights three findings central to researchers fulfilling their social responsibilities at cleanup sites. The first finding underscores the need for better understanding of stakeholders’ roles and responsibilities at sites to avoid misunderstandings. Second, the centrality of neutrality upheld by scientists limits their ability to consider and increase the social impact of their work. Third, by understanding the range of activities and knowledges being created by other stakeholders, researchers can avoid redundancies and fill a gap that can create social benefits.

Our first finding centered on the general confusion about stakeholder roles, interests and responsibilities, a pattern also reported in the Superfund literature.^58^ Using Table 6 to clarify the values and needs of different stakeholders is a critical first step to promoting social responsibility in research. Stakeholders should generally include community members, non-profit groups, public health workers, EPA workers, policy makers, small business owners and responsible parties if pertinent. Priority setting tools like the Q methodology approach could be another valuable priority setting approach.^59^ One way to understand the impact of this confusion is through the concept of therapeutic misconception wherein the participants mistakenly believe that the primary aim of clinical research is to provide therapeutic benefit, rather than to generate generalizable knowledge.^60^ In the Superfund context, communities may similarly expect researchers to intervene directly, while researchers may assume that observation and publication alone are sufficient. It is this mismatched expectation that leads to helicopter research and erodes trust.

The concept of helicopter research also referred to as parachute or parasite research, was developed mainly in the context of global research when researchers from wealthy nations conducted research on participants from lower-income countries without providing benefit to local scientists or local communities.^13,14,16^ This concept of helicopter research also applies domestically where researchers from privileged institutions or backgrounds conduct research on marginalized communities.^61^ This power dynamic is central to EJ research related to cleanup sites. Practical solutions to avoid helicopter research often center around forging non-colonial, equal and synergetic collaborations which embrace local norms and utilize local infrastructure and capacity building.^13,62^ Although our tool does mention similar relational dynamics, the tool also focuses on social benefits and impact which is more future facing.

Second, researchers were very hesitant to talk about advocacy and social benefits thinking that this would introduce subjectivity and values that would counteract their scientific norms of “objectivity” or “neutrality”. However, literature in anthropology, philosophy and sociology of science has emphasized how scientific choices are value-laden and socially constructed.^27,63-65^ The idea that science is guided only by technoscientific choices makes social responsibility challenging if not impossible. To create socially responsible research, researchers must align research with needs, values and preferences of stakeholders as exemplified in the second step of the tool. If a researcher does not believe that there is place for such values and interest, the idea of social responsibility tool is simply unapplicable. Future developments or extensions of this tool could be oriented toward helping researchers understand how values are embedded in their work so that alignment can be made.

Third, the impact of the science should be considered alongside the activities and impacts of other non-scientific activities that happen at cleanup sites. Often certain types of knowledges are already being created by other stakeholders may it be community groups of public health agencies. Conducting science about knowledge that is already developed may be redundant and wasteful. Also, collaboration with community organizations and non-profit groups that have community knowledge may be much more effective when implementation pathways are discussed.^59^ As in translational science more broadly, researchers have a responsibility to think about how they will facilitate knowledge translation.

## 5. Conclusion

Researchers should recognize that their studies at cleanup sites are not be done in a vacuum but in rich socio-cultural and ecological contexts. Assessing research at a Superfund site through a social responsibility lens will broaden and shift the emphasis on technoscientific outcomes to also consider social outcomes of value to stakeholders. The social responsibility pilot tool in this study is designed to increase the social impact of research conducted at cleanup sites. First, it would help researchers better understand the roles and responsibilities of stakeholders, manage relationships and avoid downstream misunderstandings and conflicts. Second it will help guide researchers to identify their social responsibilities to uphold relational values, enable advocacy initiatives, share expertise, take part in knowledge development, manage harms, and ensure transparency.

As previously stated, the social responsibility tool was designed in a case-specific manner to increase the social impact of science at one cleanup site, and as such, it cannot be generalized as is. It was important for our study to use a case-based approach to fully understand the complex social dynamics among stakeholders, the different values at play, and the site’s history. Given the lack of trust in EJ communities towards research and regulatory institutions, it took a significant amount of time to build trust, leading to more genuine and honest interactions and interviews. We plan to further develop and refine the tool for application at three additional cleanup sites; this should increase its potential for future generalizability. Validation and broader scale quantitative work will allow for more widespread application of the tool at other sites.

## Data Availability

Data sharing is not appropriate since its access would divulge the identity of research participants and community stakeholders. The IRB required information to be kept confidential.*
